# Governing through Prevent? Regulation and Contested Practice in State–Muslim Engagement

**DOI:** 10.1177/0038038514564437

**Published:** 2016-02

**Authors:** Therese O’Toole, Nasar Meer, Daniel Nilsson DeHanas, Stephen H Jones, Tariq Modood

**Affiliations:** University of Bristol, UK; Strathclyde University, UK; King’s College London, UK; Coventry University, UK; University of Bristol, UK

**Keywords:** counter-terrorism, Muslims, participatory governance, Prevent

## Abstract

In this article, we consider the implications of the ‘Prevent’ strand of the government’s counter-terrorism strategy for the UK state’s engagement with Muslims. We argue that the logics of Prevent have been highly problematic for state–Muslim engagement. Nevertheless, we suggest that the characterisation of state approaches to engaging Muslims as a form of discipline is incomplete without an analysis of: first, differences in practices, habits and perspectives across governance domains; second, variations in approach and implementation between levels of governance; and third, the agency of Muslims who engage with the state. Through this approach we show how attention to the situated practices of governance reveals the contested nature of governing through Prevent.

## Introduction

The previous New Labour government, through the Prevent strand of its counter-terrorism ‘CONTEST’ strategy, developed a ‘hearts and minds’ approach to counter-terrorism that emphasised partnering and engagement with Muslim communities. A dominant theme of the academic literature analysing the significance of Prevent and counter-terrorism policies has been the contention that these led to the securitisation of the state’s engagement with Muslims, with participatory initiatives being introduced with the purpose of disciplining Muslim communities, or domesticating British Islam, in the process constituting Muslims as a ‘suspect community’ (Pantazis and Pemberton, 2009) and ‘conditional citizens’ (McGhee, 2008). A number of studies (Birt, 2008; Heath-Kelly, 2013; Martin, 2014), drawing on theories of governmentality, have highlighted the disciplinary nature of state engagement with Muslims, manifested particularly in the enmeshing of security and integration policies and increasing state regulation of Muslim conduct across areas of social, cultural and religious life.

In this article, we consider the implications of Prevent for the UK state’s engagement with Muslims more broadly. In so doing, we argue that the logics of the Prevent strategy have been highly problematic for state–Muslim engagement. Nevertheless, we argue that the characterisation of state approaches to engaging with Muslims as a form of discipline is incomplete without an analysis of: first, differences in practices, habits and perspectives across governance domains; second, variations in approach and implementation between levels of governance; and third, the agency of Muslims who engage with the state. Through this approach we show how attention to the situated practices of participatory governance approaches to counter-terrorism reveals the messy and contested nature of governing through Prevent.

Our analysis of the practices of governing through Prevent is based on research carried out for the ‘Muslim participation in contemporary governance’ study (O’Toole et al., 2013).^1^ This study examined policies and practices of state engagement with Muslims, their implications for Muslim civil society organisations and Muslim responses to governing agendas, at national and local levels from 1997 to 2013. In particular, we analysed three policy domains where government has increasingly recognised or engaged with Muslims: equalities and diversity policies where recognition of religious, and Muslim, difference and identities has come increasingly to the fore; participatory approaches to welfare delivery and service provision where faith-based, including Muslim, organisations have emerged as key partners or stakeholders; and security and counter-terrorism, which has involved a Muslim community engagement strand as a key element of the Prevent strategy. Our study was based on policy analysis and 112 semi-structured interviews with government and Muslim civil society actors working at the national level and in three local case-study areas of Birmingham, Leicester and Tower Hamlets. Our sample included representatives from national civil society organisations active on issues relating to Muslims (e.g. Cordoba Foundation, Muslim Council of Britain (MCB), An-Nisa Society and Quilliam), civil servants and political advisors in government departments, including the Office for Security and Counter Terrorism (OSCT) and Department for Communities and Local Government (DCLG), politicians and local government, faith and Muslim civil society actors in Birmingham, Leicester and Tower Hamlets.^2^ Our interviews featured generic questions on the nature and quality of state–Muslim engagement, supplemented by questions tailored to our interviewees’ areas of work and expertise.^3^ Our data enable us to consider the logics and practices of state engagement with Muslims across policy arenas, and at different levels of governance, to address the question: what impact did the state’s engagement with Muslims through the prism of counter-terrorism have on its engagement with Muslims more broadly?

In the next section we outline the core components of the Prevent strategy before turning to read the implications of this strategy through two conceptualisations of governance through which Prevent has been interpreted: one as a disciplinary mode of regulation and the other as contested practice.

## Engagement through Prevent

The Prevent strategy that was unveiled in 2007 in response to the 2005 London bombings was framed as a ‘hearts and minds’ approach to countering al-Qaeda-inspired domestic terrorism. The provenance of Prevent rests in a broader strategy cumulatively developed since 9/11. Known as CONTEST, and launched in 2003, this contained four components including: Pursue (surveillance and detection); Prepare (civil emergency contingency planning); Protect (domestic security); and Prevent (tackling ‘radicalisation’). It is this last objective that has most overtly sought the participation of Muslim communities.

The Prevent agenda entailed significant shifts in the government’s relations with Muslim religious, civil and community organisations. ‘Preventing Violent Extremism’ (PVE) or ‘Prevent’ initiatives focused on Muslim community engagement and capacity-building, with a particular focus on theological, youth, women’s and counter-radicalisation projects. Thus, the New Labour government set out to develop theologically based counter-narratives to al-Qaeda ideology by funding the Radical Middle Way project, and facilitating the creation of the Mosques and Imams National Advisory Board (MINAB) to create a UK-based system of mosque regulation. It reconfigured the mechanisms for the representation of Muslims vis-a-vis government, with the creation of the National Muslim Women’s Advisory Group and the Young Muslims Advisory Group in 2008, which bypassed existing Muslim representative structures to enable the government to engage directly with Muslim women and youth. Through such means, as the Prevent strategy document declared, the government’s aim was to: ‘fundamentally rebalance our engagement’ (DCLG, 2007a: 9). Prevent initiatives were facilitated by substantial funding. There were three government departments charged with delivering Prevent, each holding their own budget: the Home Office, particularly the OSCT; the DCLG; and the Foreign and Commonwealth Office. Between 2008 and 2011, their combined Prevent funding came to £186,760 million.^4^

Tensions in the logics of engagement with Muslims under Prevent were evident from the start: on the one hand, the DCLG’s PVE Guidance noted among Muslims there was ‘a tiny minority who oppose tolerance and diversity’, but stated nevertheless that a ‘key measure of success will be demonstrable changes in attitudes among Muslims’ (DCLG, 2007b: 7). Fairly soon after its inception, Prevent was subject to extensive criticism. In particular, critics argued that the focus on Muslims, the approach to community engagement through the prism of counter-terrorism and the overlap between Prevent and Community Cohesion policies securitised state engagement with Muslims (Thomas, 2012) and cast Muslims as a ‘suspect community’ (Pantazis and Pemberton, 2009). Kundnani’s (2009) report, *Spooked*, fuelled such perceptions by citing reports of Muslim youth and community workers being approached by intelligence services to provide information about the communities with whom they worked. This was denied by the government, with a Home Office Minister, Vernon Coaker, protesting: ‘Accusations that Prevent is about spying on people or criminalising vulnerable communities are simply untrue, and only jeopardise the vital work of Prevent to work with communities and keep vulnerable communities safe from radicalisers’ (cited in Birt, 2009: 56). This claim was undermined however by subsequent statements by the architect of the government’s counter-terrorism strategy, David Omand, who in his testimony to the All Party Parliament Group on Homeland Security in 2010 observed:

[Y]ou can’t divide government in two, into those people that go around spying on the population, and there are another lot of people going round to [i.e. engaging with] the population and they just don’t talk to each other. It just simply doesn’t work like that. (cited in Thomas, 2012: 118)

Suspicions about the rationale for Muslim engagement that was being pursued under Prevent proved difficult to dislodge. As a House of Commons Select Committee acknowledged, Prevent was widely perceived as a spying programme, with one witness characterising Prevent as ‘Pursue in sheep’s clothing’ (House of Commons, 2010: 8). The view that Prevent created highly securitised forms of community engagement appeared to be corroborated by the role of the OSCT in delivering Prevent, and the organisational overlap between security services and community engagement teams that occurred at the local level. In Birmingham, for instance, the Prevent programme there was led by a counter-terrorism police officer who had been seconded from the regional Counter Terrorism Unit (CTU) into the city council’s Equalities Division. A local community activist in our study commented:

Locally we’ve had […] a controversial issue with a police officer […] who was seconded into the Council. I can remember clearly very early on, members of the youth inclusion project […] said that it increased their own suspicions of why he was involved in it. […] And the very first question they were posing to [him] was, ‘This is security-led, intelligence-led. Otherwise you wouldn’t be here.’

Husband and Alam (2011) point out that the multi-agency nature of its delivery meant that on the ground Prevent came to permeate a wide range of policy areas. Consequently, as many argue, a host of policies, including integration, Cohesion and civil renewal policies, became problematically linked to counter-terrorism (Thomas, 2012). Many respondents in our study concurred with the belief that engagement conducted through Prevent was securitised, with concerns about political extremism displacing other issues. As one former adviser to the DCLG, Alveena Malik, told us: ‘equality and diversity wasn’t seen as an issue. It wasn’t seen as certainly a solution. It was around how do we deradicalise?’ The contention that engagement through Prevent constituted a limited offer of participation was widely substantiated by respondents in our study. As Alveena Malik remarked ‘there was this burden of responsibility and blame that we had to deal with which I found really difficult, which I rejected […] those of us who didn’t toe the line, we were shunned and silenced.’ Birt (2009: 54) concludes that consequently within government there was an ‘overemphasis upon counter-terrorism without engaging Muslims as citizens’.

## Prevent as a Mode of Disciplinary Governance

A significant body of critical literature has developed analysing the various ways in which ‘hard security’ measures, such as Schedule Seven and Section 44^5^ policing powers enabled by prevention of terrorism legislation, were supplemented by an array of ‘soft security’ measures and discursive interventions – exemplified by Prevent – that constituted a developing array of managerial techniques that sought to know, reform and discipline British Muslims. A number of Foucauldian-inspired readings have focused on the routine and pre-emptive ways in which government sought to inculcate discipline and ‘self-governance’ among Muslims (Birt, 2008; Heath-Kelly, 2013; McGhee, 2008; Martin, 2014), in Foucauldian terms: bringing together ‘practices of the self with those of the practices of government’ (Dean, 1997: 208). Mavelli (2013: 161) argues that state responses to terrorism went beyond ‘exceptional measures’ and manifested themselves in a wholesale reorganising of ‘everyday “normal” political and bureaucratic rationalities’. Drawing on actuarial techniques of governance (Miller and Rose, 2008), Heath-Kelly (2013) suggests that government adopted a pre-emptive approach to managing security risks within the counter-terrorism paradigm in which Muslims were constituted as simultaneously ‘at risk’ (that is vulnerable to radicalisation) and ‘risky’ (posing a security threat). This entailed a series of wide-ranging interventions in Muslim religious, social and civil structures, with the aim of reforming, managing, regulating and ‘disciplining’ Muslim conduct. According to Heath-Kelly (2013: 395), what developed through Prevent was ‘a more-or-less cohesive project of risk knowledge which [was] deployed to render terrorism pre-emptively governable’.

Birt (2008: 27) argues that through Prevent, government developed an array of mechanisms for the disciplining of Muslim subjects, including: the deployment of a set of reductive distinctions between good/bad, moderate/extremist Muslims, creating a limited repertoire of subject positions for Muslims (and see McGhee, 2008); as well as attempts to mobilise the Muslim community as a whole in combating extremist ideology; the reform of ‘conservative’ Muslim practices and Muslim institutions; and the promotion of mainstream, liberal Islam. Despite this, Birt (2008: 28–29) observes Muslims have been ‘successful in disrupting the application of governmentality’ by acting above or below the nation-state – through engagement in either autonomous grass-roots organisations or global Muslim publics. Brown (2010) highlights a range of initiatives by British Muslims (such as the Muslim lifestyle and current affairs magazine *Emel*) that sought to present alternative perspectives on British Muslim identities and Islam in order to resist the subject positions offered to them by state discourses on Muslims, integration and security – although these too were pursued outside of arenas of governance. Such perspectives seem to suggest that there has been little scope for Muslim actors to participate as autonomous or effective actors *within* governance.

## Prevent and Governance in Practice

The logics and aims of Prevent have been highly problematic in terms of their positioning of Muslims as a security risk and poorly integrated into British society and the limited, and limiting, offers of engagement that have characterised participatory initiatives conducted under Prevent. However, we contend that the governance of Muslims through Prevent in *practice* has been less complete, and more contested, than many studies have allowed. Our account of ‘governance’ defines it in terms of ‘new processes of horizontal decision making and collaborative modes of governing between public, private, voluntary and community actors’ (Griggs et al., 2014: 2). This is distinct from ‘government’ that is typified by vertically integrated forms of decision making by public officials and political professionals. It is also distinct from the more expansive notion of governance that is prevalent in the governmentality literature, which focuses on a wide variety of state and extra-state agencies that seek to work on the conduct of individuals. In our critique of prevailing accounts of Prevent and governance, we highlight three conceptual and methodological issues that inform our perspective on its implications for state engagement with Muslims.

First, many studies of Prevent have focused on its discursive underpinnings through textual analysis of policy documents. More generally, McKee (2009: 473) identifies a tendency of many Foucauldian-inspired studies of governmentality to reduce Foucault’s interest in the ‘specific and concrete “art of governing”’ to ‘the rationales of governing as manifest in key (government) documents’. While the discursive dimensions of Prevent, and the logics of governing and Muslim engagement that are expressed in policy documents, *should* be analysed, we suggest there has been a neglect within the literature on Prevent of the material practices of governing (McKee, 2009: 473). With a few notable exceptions (Fussey, 2013; Husband and Alam, 2011; Lowndes and Thorp, 2010; Thomas, 2012), studies of Prevent and counter-terrorism policy have infrequently engaged with the practices, and conflicts, that have underscored the development and implementation of policy texts. Yet, and crucially, as McKee (2009: 474) argues, ‘it is a mistake to ‘read off’ consequences from governmental ambitions […] for it cannot be assumed that […] power always realises its effects’. This is an important observation, and as our data show, through analysis of how actors interpreted, responded to or implemented Prevent, we see that the *practices* of Prevent did not always cohere with the *aims* of Prevent that were expressed in various policy documents.

Second, many studies overemphasise the unity and coherence of governing strategies. Yet, as McKee (2009: 474) notes, ‘governmental programmes and strategies are themselves internally contradictory, continuously changing and capable of mutation’. The dispersal of governing functions to a range of governance networks and partnerships over the last two decades, moreover, has created a highly differentiated governance terrain, with different government departments, networks and levels operating with different practices of governance. As ‘political fields’, the domains of governance are typically, following Swarz (2003: 151), arenas ‘of conflict over the definition and implementation of public policies that are struggled over by political professionals’. The implications of this are that governance is increasingly dispersed and often internally contradictory and contested, requiring study of the range of practices of governing across different governance domains. Fussey’s (2013: 356) analysis of counter-terrorism policy is notable in drawing on a broader account of governmentality theory, in particular utilising Foucault’s theorisation of security and the specific loci of security practices as an alternative to a reified panoptical state, to provide a conceptual tool to analyse the diversity and heterogeneity to be found within counter-terrorism practice. Taking this approach, he contends that ‘heterogeneity and conflict’ characterise ‘the landscape of security practice’, particularly as it ‘becomes increasingly populated with diverse actors and agencies’ (2013: 352). Consequently, he questions both ‘the extent to which ambitions for control are realised and the degree of coherency within coalitions of [counter-terrorism] practice’ (2013: 356). Our own analysis concurs with this more heterogeneous reading of practices of governing through Prevent.

Third, inspired by a Foucauldian disciplinary analytical frame, studies of Prevent have tended to focus on governing power at the expense of agency. Despite the availability of a theoretical account of counter-power within Foucault’s work, Bevir (2011: 462) notes that ‘concrete studies of governmentality rarely examine agency as a source of discourses or as evidenced in specific instances of counter power’. This tendency is also noted by McKee (2009: 473–474), who suggests ‘this preference to disregard messy empirical actualities results in a fundamental inability to account for why the governance subject, constituted through discourse, fails to turn up in practice’. Yet, as McKee acknowledges, Foucauldian theoretical frameworks can, in Fussey’s (2013: 359) terms, facilitate ‘an appreciation of the heterogeneity of control, incorporating diverse state and non-state actors, and the way in which security practices aspire neither towards total coercion or control’. We argue, however, that much of the ‘disciplinary’ analytic that has come to characterise analyses of Prevent does not do justice to this fuller agency-incorporating sociology of governance. The need for a more complete account becomes particularly evident in the dispersed and multi-layered delivery of Prevent and in its reception across different levels of government and within Muslim civil society. A more practice-based approach to analysing state–citizen relations within governance spaces can reveal the possibilities for citizens to effect more autonomous agendas than those necessarily marked out for them by governors. Taking such an approach, Cornwall and Coelho’s (2006: 11) account of the responses of citizens who are invited into participatory governance spaces proposes that they should be seen as potentially ‘spaces for change’, because although they are ‘invited spaces’, that are ‘framed by those who create them, and infused with power relations and cultures of interaction carried into them from other spaces’, they are ‘also spaces of possibility, in which power takes a more productive and positive form’. Proceeding with this understanding of governance opens up theoretically the possibility that actors within governance spaces may adapt or change formal rules of governance in ways which may depart from institutional design – sometimes with unintended consequences – necessitating ‘a larger role for contingency in understanding governance’ (Griggs et al., 2014: 9). Such an approach recognises the potential for the exercise of agency by different actors in reinterpreting, appropriating, contesting or resisting governance practices.

Through a practice-oriented analysis of how Prevent was formulated, received and implemented, we advance an understanding of its implications for state–Muslim engagement as contested practice. In common with Fussey’s (2013) account of counter-terrorism surveillance measures, we show how Prevent was subject to contradictory and inconsistent logics and outcomes within government, and encountered modification, challenge and resistance – both from state actors at national and local levels and from Muslim actors acting within and outside of governance arenas. The following sections set out the empirical basis to our argument. We begin by showing some of the contradictory logics of Prevent across government departments, and how these were met and contested by local authorities in different localities, before addressing responses by Muslim actors to the articulation and implementation of Prevent.

## Logics and Practices of Engagement across Government

One recurring issue that emerged in the critique of Prevent under New Labour was the blurring between Prevent and Community Cohesion policies. This was underpinned ideologically by the conviction that Cohesion was necessary for Prevent, and operationally by the overlap between the OSCT and the DCLG in the delivery of Prevent. Thus, each held its own Prevent budget, while the DCLG also assumed responsibility for the delivery of Community Cohesion policy. Actors within the OSCT and the DCLG had, however, somewhat different conceptualisations of Muslim community engagement through Prevent (O’Toole et al., 2012). The view within the DCLG tended to see faith communities as resources in the delivery of Cohesion and welfare (Dinham and Lowndes, 2008), emphasising the need to identify key stakeholders and engage ‘hard-to-reach’ groups within governance networks. As one senior adviser to the DCLG, Maqsood Ahmed, explained to us: ‘I was involved in the Prevent and when I say Prevent, it was less to do with the counter-terrorism, more to do with how do we establish connection with the Muslim community; how do we capacity build in the community’, and in ways which went beyond ‘the “usual suspects” who are always on the Government table’. In contrast, a senior civil servant in the OSCT told us:

In the early days, I had no idea that this job would involve community engagement. There were a few reasons for that: one, no-one had told me, and two, the organisation was new and we hadn’t designed it into the principles.

There was evidence of a turf-war between these departments in the delivery of Prevent, as the senior OSCT civil servant suggested:

The DCLG regarded it as their job to have those contacts: to the point where they didn’t particularly want us to have them. I think if truth be told, they were also slightly apprehensive that we would come in with size 12 security boots and sort of damage the contacts that they were creating.

Ultimately, he suggested, the OSCT had used its power and resources to dominate the interpretation and delivery of Prevent:

Because we arrived in a rather security-like way with a very determined delivery plan, occasionally people were just run off the court. They didn’t have as much money. They didn’t, frankly, have as much drive. They didn’t quite know what they were doing. And it was hard. So what happened was Prevent took over Cohesion.

Consequently, a fairly underdeveloped model of community engagement became increasingly dominated by a more focused security agenda. According to the OSCT officer: ‘We made a fundamental mistake three years ago with Prevent. I thought that we would be able to place Prevent on top of a rich seam of dialogue with Muslim communities. My mistake was that that seam didn’t exist.’ Echoing this analysis, a former Secretary of State at the DCLG, John Denham, revealed to us: ‘I found in the CLG, after some very rigorous examinations with officials, that there was no understood model of how Prevent was meant to work.’ This left Prevent prone to domination by security actors and issues – but rather than driven by a unified, coherent governing project, this was an outcome of internal struggles between political professionals over its definition and implementation.

This issue of overlap between Prevent and Cohesion, and the consequences of this for Cohesion, came to the fore in a House of Commons enquiry into Prevent, which recommended their future separation. This critique informed the incoming Coalition government’s review of Prevent in 2010, which acknowledged that overlap between the two had ‘led to accusations that the government’s interest in Muslim communities is related only to the risk of terrorism’ (Home Office, 2010). Subsequently, the revised Prevent strategy that was announced in June 2011 stipulated that Prevent and Cohesion would henceforth be separated – with the Home Office leading Prevent and the DCLG leading Cohesion policy – although as we discuss later, this separation is unlikely to be maintained in practice.

A second issue identified by the 2011 revised Prevent strategy related to the government’s conceptualisation of ‘extremism’, and its engagement with ‘moderate’ and ‘extremist’ Muslim groups and actors. New Labour’s position on this question shifted. On the one hand, they condemned ‘intolerant’ and extremist perspectives while seeking out and supporting ‘moderate’, ‘mainstream’ Muslims. On the other hand, they attempted to differentiate between ‘extremist’ and ‘violent extremist’ positions in order to engage pragmatically with non-violent ‘extremist’ Islamist organisations (Birt, 2009: 54). One former Home Secretary (2004–2006), Charles Clarke, in interview, confirmed that within the New Labour government there was little clarity or consensus on this issue:

there was not a clear approach to what needed to be done, but there was a wide range of different views, there was confusion over some leaders of some of the communities and in particular, confusion about the extent to which we should, in any sense, compromise with some of the forces which have deep, deep, deep roots and a lack of appreciation of the nature of our democratic society in which we live […] I do think it was an issue where we didn’t have a coherence about what we thought was the right way of dealing with this question.

This lack of clarity was confirmed by a former policy adviser to the DCLG, Francis Davis, who commented that there was ‘no real kind of clear basis upon which one can decide who’s in and who’s out. So you get Hazel [Blears] throwing MCB^6^ out and you get John [Denham] and the team working very hard to get them back in.’ In his speech to the Munich Security Conference in 2011, David Cameron (2011) signalled a hardened stance on this issue, declaring: ‘instead of ignoring this extremist ideology, we – as governments and as societies – have got to confront it, in all its forms’. Cameron’s ‘muscular liberalism’ has ostensibly prevailed in the new Prevent strategy in its stipulation that government will no longer engage with ‘extremists’, including non-violent extremists (that is, non-violent Islamists or Salafis), who do not subscribe to core liberal values (Home Office, 2011: 23–24). This stance, however, has not been shared by other Ministers in the Coalition, with divisions crossing party lines (Oborne, 2011), nor has it been shared by key civil servants within the OSCT, as one senior officer told us at the time of the Prevent review:

I don’t want to be dealing with extremism per se anywhere. I don’t think it’s right for us as a counter-terrorism team to be doing that. […] Extremism isn’t illegal in this country and we’re not the right people to deal with that. We must get involved where extremism is leading people on the road to terrorism.

Indeed, our study found there was significant scepticism over whether this stipulation *can* be implemented. As one Muslim adviser to the Home Office recounted to us in late 2012:

I just went to a meeting two weeks ago and the Home Office is still working with those groups. […] if you want to reach hardline Salafi communities, you have to work with hardline Salafi people, that’s just the way it is. You’re not going to reach them through cuddly Sufis. There’s a sense of *realpolitik* about this whole thing.

Such conflicts over core issues relating to the rationale of Prevent contributed to a sense that Prevent was unfocused (Birt, 2009), and consequently, less cohesive than often imagined. This was a charge levelled particularly at New Labour’s strategy. While the Coalition’s strategy lays out a stronger line on eschewing non-liberal ‘extremist’ groups and seeks to mobilise a range of front-line services (including charities, universities and health services) in pre-emptively responding to extremism, in practice this stance is also less coherent than it appears, as we discuss below.

## Local Reception and Implementation of Prevent

Perceptions of the unfocused nature of Prevent under Labour were very prevalent at the local level. In part, this was encouraged by the somewhat open-ended nature of Prevent guidance, which gave local authorities leeway to interpret and implement Prevent as they saw fit. As John Denham (2013), later reflected: ‘With no clear national guidance on forging allies against terrorism, mistakes were inevitable […] Despite this, Prevent did good work in areas where people worked through the challenges for themselves’. Local Authorities often reworked Prevent to fit local priorities, and, as Lowndes and Thorp (2010: 124) show, local contexts were important in shaping the outcomes of Prevent – notwithstanding ‘the intensity of national debate and the apparent “top–down” character of the agenda’. Lowndes and Thorp (2010: 124) also focus on the ‘creative and often surprising interplay’ between central strategies and local agency in their study of the implementation of the Prevent Pathfinder programme of 2007–2008 in three Midlands cities, which served as the pilot phase of the Prevent programme that followed. They found that models of community engagement varied significantly across the three cities – from community safety, community cohesion to community development approaches – reflecting their different logics, histories and pre-existing structures of community engagement.

Prevent was met with considerable opposition by many local authorities, as other studies have documented (Thomas, 2012). In particular, several local authorities in areas that were targeted for Prevent funding were reluctant to adopt National Indicator 35 (NI35) – the government’s national assessment framework for monitoring resilience to violent extremism. As the minutes of a Local Government Association (LGA) meeting of 2008 recorded: ‘Local authorities are reluctant to pick up the indicator because the term “violent extremism” could alienate communities, undermining cohesion work and are extremely cautious about making public statements around PVE’ (cited in Khan, 2009: 8). Husband and Alam’s (2011) study of Prevent in West Yorkshire found that it encountered ‘strong resistance’ from local authority personnel, with one senior councillor in their study denouncing Prevent as ‘racist’ (2011: 147). In Bradford, the Council initially refused to accept Prevent funding, but did eventually although, as a former Conservative Bradford council leader, Kris Hopkins, explained to us, not to implement Prevent objectives:

And so you ended up with a situation where we [Bradford Council] were out there […] trying to gain the confidence of a very fragile community^7^ through PR interventions around Community Cohesion, and at the same time we were being asked to be an arm of the security services, to respond under the direction of the NI35 directives. And we just refused to play. And they said well you can’t have the Prevent money unless you play, so we said […] keep your Prevent money, we’ll spend our own reserves to do more Community Cohesion work. And eventually they gave us the Prevent money anyway.

Across our three local case study areas of Leicester, Tower Hamlets and Birmingham, we found considerable variation in the ways in which Prevent was conceptualised, received and implemented under New Labour. Significantly, we find that variation continues under the Coalition’s revised Prevent strategy – despite attempts to enforce a more focused and centralised approach.

In Leicester, when Prevent was launched in 2007, the local council refused to accept the terminology of Prevent, rebranding it ‘Mainstreaming Moderation’. Largely because it is host to a substantial Gujarati population with significant numbers of Hindus, Sikhs and Muslims, local governance in Leicester has since the 1990s had a strong multi-faith ethos, which Prevent’s exclusive focus on Muslims placed at risk (Open Society Institute, 2010). To mitigate this, the strategy was reconceived to include all forms of extremism – despite local perceptions of opposition to this from central government. In 2011, the elected mayor went further, refusing to accept a Home Office funded Prevent Officer within the Council. This led to Prevent being co-ordinated by a local Church-led multi-faith centre, St Philips Centre, in partnership with the city’s Muslim umbrella body, the Federation of Muslim Organisations (St Philip’s Centre, 2012). St Philip’s Centre is one of the more significant non-governmental bodies in Leicester. With a background in Cohesion work, it currently delivers the Coalition’s flagship integration programme, Near Neighbours. It is thus unlikely that Prevent and Cohesion can or will be separated in Leicester as the new Prevent strategy directs. Indeed, the Centre’s Director maintained in interview: ‘if you are going to tackle issues of extremism you have actually got to address issues of integration as well. The reality is they do belong together’, while the Deputy Director observed: ‘St Phillip’s […] is an organisation which has developed its reputation on interfaith relationships. The Home Office knows that and is happy with that. What other option did they have, you could argue!’ In practice, then, Prevent in Leicester maintains a close relationship with Cohesion.

In Tower Hamlets, the local council was initially cautious, but not hostile, towards Prevent. Its implementation of Prevent tended to place strong emphasis on projects with a Community Cohesion orientation, with 24 out of 28 projects focused on broad Cohesion aims rather than more security-related issues (Iacopini et al., 2011). Following the launch of the revised Prevent strategy, projects proposed by Tower Hamlets in 2012 were rejected by the Home Office as too ‘Cohesion oriented’. Nevertheless, Tower Hamlets council has been pursuing its own locally shaped projects – such as the ‘No Place for Hate’ programme, which excludes extremist preachers, but emphasises local cohesion in the face of threats from the far right – and therefore overlap remains. Significantly, Tower Hamlets also opted out of the Channel programme^8^ – setting up its own programme that is accountable to local authority and policing structures rather than directly to SO15 (the counter-terrorism command within the Metropolitan Police). Despite central government’s bar on working with Islamists, in Tower Hamlets the East London Mosque (ELM), often vilified from outside as an Islamist mosque, maintains its position as a key institution locally. It is deeply embedded in local governance networks and the largest non-governmental provider of local services. Thus, it would be difficult, indeed counter-productive, for local government to disengage from the ELM.

In Birmingham, the city council initially implemented Prevent with cooperation from some Muslim organisations. But, perceptions that Prevent was police-led arose quite early on, not least, as noted above, because of the secondment of a counter-terrorism police officer into the council’s equalities division to lead Prevent. By 2010, suspicion towards Prevent had intensified as a consequence of ‘Project Champion’. While not itself a Prevent initiative, Project Champion did much to undermine the implementation of Prevent in the city. Project Champion was a police surveillance operation involving installation of 216 CCTV and ANPR (overt and covert) cameras in two areas of Muslim settlement, creating a ‘surveillance ring’ around these areas. Importantly, the counter-terrorism purpose of the cameras was concealed from local residents, and the cameras were badged as a ‘crime safety initiative’ with little, and deeply flawed, community consultation. Furthermore, Fussey (2013) found serious divergences among the coalition of professionals involved in Project Champion, in terms of their knowledge of and support for the project. The true purpose of Project Champion was revealed by civil society campaigners (Jolly, 2010) with damaging implications for Prevent. By the time Prevent was revised and re-launched in 2011, there were very high levels of suspicion towards Prevent, with few projects underway, such that in the words of one of our interviewees, ‘Prevent is dead in this city.’

At the local level, then, there were differing perceptions of Prevent, with implications for its reception, delivery and impact, with local actors modifying, challenging and resisting Prevent, such that in practice it varied from one locality to another and often diverged from centrally articulated policy.

## Muslim Civil Society Responses to Prevent

A third much-neglected area in the study of the implications of Prevent has been the responses of Muslim civil society actors to the models of community engagement developed through Prevent. Our study uncovered a range of responses, from qualified cooperation, to appropriation of aspects of Prevent (including funding) for more autonomous objectives, to participating after renegotiating the terms of engagement offered by government, to challenge, protest and exit. Many actors shifted positions across these range of responses.

A very common response of Muslim organisations and actors was a simple refusal to engage with Prevent. Birt (2009), for example, reports that the Lancashire Council of Mosques refused funding because the scope of Contest 2 was too broad in its focus on Muslims in general. This objection was a very common one, and increasingly augmented by widespread suspicion of Prevent as a surveillance programme (Husband and Alam, 2011). Thus, even some Muslims who might have welcomed the funding that Prevent provided were dissuaded from engaging with Prevent because of its stigmatising associations and fears about its covert aims. There were also Muslim groups and organisations that had engaged with the relatively more open-ended Pathfinder programme in 2007–2008, but decided against participating in any further Prevent activity – despite the possibilities that such a tranche of funding might enable. As one member of a Muslim organisation, the Cordoba Foundation in London, which had been a recipient of Prevent funding, commented:

I won’t touch [Prevent funding] with a two metre bar and no-one will. No-one of any decency will and that’s a big problem. That’s a huge problem because we ought to be [making use of funding]. This is public funds for the public good. Why aren’t we using it? We can’t because now it’s poisoned.

Some who had engaged during the Pathfinder year, had done so with reluctance, but had also sought to renegotiate the terms of their engagement, as Humera Khan, in discussing the involvement of her community organisation, An-Nisa Society, explained:

we took money in the Pathfinder year. We were persuaded by our youth service and diversity team, because they didn’t have anybody else who had the ability to run a project. […] We said we would only do it on the condition that it’s not sold as a Prevent project. We’re not going to do Prevent work, we’re going to do community development […] we want it to be as a basis to start a dialogue with you as a council.

Subsequently, however, An-Nisa refused to engage any further with Prevent, and published a highly critical report arguing that Prevent was flawed on a number of grounds, including: ‘its targeting of the whole Muslim community as potential terrorists’; ‘the fusion of counter terrorism with community cohesion and community development initiatives’; ‘the mainstreaming of Prevent in the core services of local councils’; its ‘heavy surveillance focus’, its ‘confusing and unclear’ strategy; the role of ‘ill equipped councils’ in attempting to intervene ‘in a highly sensitive area’; the loss of credibility and trust incurred by those grass-roots Muslim groups who accepted Prevent funding; and the lack of concerted government action on tackling social exclusion among Muslims (Khan, 2009: 4–5).

Several organisations used their expertise and positions as gatekeepers to particular groups as negotiation tools to insist on their own terms of engagement. As Abdul Haqq Baker, the organiser of the STREET (Strategy to Reach Empower and Educate Teenagers) project in Brixton explained:

STREET, I think, became a very powerful tool to show the effective engagement and partnership, especially partnership, with government entities, whether they be local or central, and NGO institutions was possible. But the key area here was that it was negotiated on equal terms. There were some terms that I would not accept from local partners […] e.g. the police and other statutory organisations saying you need to inform them and provide reports on your target audience. I said I won’t do that, that’s not going to happen, and I’m prepared to walk away from any agreement on that basis, because of the confidentiality, because of the credibility that we’ve got with such individuals.

In other areas, there were cases of more open confrontation and protest against certain counter-terrorism measures. In Birmingham, this was particularly manifested in the campaign against ‘Project Champion’ that adopted the name Birmingham Against Spy Cameras (BASC). This was a concerted, successful grass-roots campaign by Muslim and non-Muslim activists to identify and dismantle the cameras. Their action forced a public apology from West Midlands Police, followed by the hooding of the cameras and ultimately their removal (and see Fussey, 2013).

A number of activists in our study who had worked with the government on Prevent had voiced their objections to the logics or practices of Prevent, with many withdrawing from engagement with Prevent or Channel or other counter-terrorism initiatives that sought Muslim cooperation. We found a range of exit strategies. For instance, one Muslim mentor in Birmingham who had been working with the OSCT on the Channel programme quietly withdrew from the scheme because he felt unable to sign up to their ‘Individual Values’ form.^9^ Other exit strategies were more public and openly challenging. An-Nisa’s exit from Prevent was, as noted above, accompanied by the publication of a highly critical report. Similarly, a prominent member of the government-established National Muslim Women’s Advisory Group, Shaista Gohir, resigned from the group and issued a public denunciation of the Brown government’s approach to engaging with Muslim women (Gohir, 2010). We found instances of actors exiting Prevent and attempting to establish their own alternative initiatives, including an attempt by community activists in Birmingham to establish an alternative, autonomous Muslim self-organised Prevent programme, which eschewed government funding or involvement and was distinctive in its critical focus on both UK foreign policy *and* al-Qaeda’s ideology and tactics.

## Conclusion

In this article, we have focused on the implications of state engagement with Muslims through the prism of Prevent. We opened by identifying tensions between two conceptualisations of governance: one as a disciplinary mode of regulation and the other as contested practice. In the literature, the former is pervasive. Undeniably, much of the logic of Prevent has been disciplinary in its aims and as such, this has had enormous symbolic and discursive effect. Nevertheless, we suggest that the latter is also in operation, as attending empirically to how different actors responded to Prevent reveals: its policy logics were often inconsistent, while its practices were messier and more contested than many studies have allowed. Engaging with ideas of practice opens up the possibility that while the state may initiate engagement in problematic ways and with limited offers of participation, actors across governance arenas may not necessarily comply with these logics of engagement. As such, we contest the view that Prevent can be seen straightforwardly as a form of discipline given its contradictory, incoherent, and contested practice.

Nevertheless, Prevent did underwrite what Archer (2009: 332) cites as a ‘politics of unease’ around Muslims in British society, which, following Huysmans and Buonfino, he describes as a ‘patchwork of insecurities that facilitate the policy exchange of fears and beliefs’ across a range of governance domains. Such ‘policy exchange’ occurred because there was, as Birt (2009: 57) points out, ‘an inadequate firewall’ between Prevent and Pursue, and as Alam and Husband (2013) argue, the multi-agency nature of delivery enabled such permeation, while prior to 2010 the operational overlap between Prevent and Cohesion carried security concerns over into the implementation of Cohesion and integration strategies. As our data show, in these circumstances, where there were asymmetric relations between the OSCT and the DCLG, security issues overrode other concerns and approaches – but this was not based necessarily on a consensual understanding of Prevent. Further, we have argued, Prevent was rejected, modified, contested and opposed by Muslim civil society and governance actors on the ground. Under the new strategy, Prevent and Cohesion have, in policy terms at least, been separated (although we suggested in practice this separation is incomplete). However, there is potential for Prevent to leach into other policy areas due to the new strategy’s focus on institutions and sectors and its goal to charge front-line personnel in schools, universities, health services and charities with Prevent delivery. While it is likely that Prevent will continue to be patchy, contested and/or resisted by governance actors, professionals^10^ and citizens within those sectors and institutions, it has the potential to facilitate ‘a policy exchange of fears and beliefs’ across governance domains and entrench further a ‘politics of unease’ about Muslims in British society.

## Notes

1. This project was part of the AHRC/ESRC *Religion and Society* programme: http://www.religionandsociety.org.uk/.

2. For a full list of interviewees see O’Toole et al. (2013: Appendix 1).

3. The study operated a strategy of differential disclosure, where respondents were given the option of being reported by name, general role, anonymously or not reported/cited directly at all, and in relation to all or parts of their interview data.

4. Between 2008 and 2011, the DCLG budget for Prevent was £66.760 million according to a detailed response by the DCLG to a Freedom of Information request in 2011: see https://www.gov.uk/government/publications/prevent-funding. Between 2009 and 2011, Home Office funding came to £84 million, while FCO funding came to £36 million: see Home Office (2011).

5. These provisions grant the authorities the power to stop, search and hold individuals without the need to provide grounds.

6. Founded in 1997, the Muslim Council of Britain (MCB) is the UK’s largest Muslim umbrella organisation.

7. This is a reference to the 2001 disturbances in Bradford (and the nearby towns of Burnley and Oldham), which were characterised as ethnic conflicts that stemmed from patterns of segregation among Asian and white communities.

8. Channel is a multi-agency programme that aims to identify individuals ‘at risk from radicalisation’ and develop support plans for them – often involving mentors. See the government’s Channel guidance, available at: https://www.gov.uk/government/uploads/system/uploads/attachment_data/file/118194/channel-guidance.pdf.

9. Mentors participating in Channel were required to sign a Statement of Individual Values form that included questions on the putative mentor’s attitudes towards equalities, democracy, religious tolerance, homosexuality, ethnic and religious pluralism, violent protest and ‘support for armed resistance against British troops overseas’.

10. For example, in response to a new policy ‘issued by NHS England to all health authorities’ stipulating that ‘GP practices must train a lead member of staff to recognise patients who could become or have become linked to terror groups’, the chair of the Royal College of General Practitioners stated: ‘It is completely disproportionate and a poor use of GP resources and time. It is effectively asking GPs to be a government intelligence agency’ (Johnston, 2014).
